# Emotional Intelligence as a Protective Factor against Victimization in School Bullying

**DOI:** 10.3390/ijerph17249406

**Published:** 2020-12-15

**Authors:** Benito León-del-Barco, Santiago Mendo Lázaro, María-Isabel Polo-del-Río, Víctor-María López-Ramos

**Affiliations:** Department of Psychology, Faculty of Teacher Training College, University of Extremadura, 10071 Cáceres, Spain; mabelpdrio@unex.es (M.-I.P.-d.-R.); vmlopez@unex.es (V.-M.L.-R.)

**Keywords:** school bullying, victim, emotional intelligence, primary education, pupils

## Abstract

Previous research has identified the main predictors of being a victim of school bullying. This study focused on the phenomenon of school bullying and its relationship with self-perceived emotional intelligence. The main aim was to analyze the mediating effect of emotional attention, clarity, and repair in relation to school victimization. The sample was made up of 822 primary school pupils from 10 public schools. Data were collected through self-reports, exploring the profile of victims of school bullying, and the dimensions of self-perceived emotional intelligence (PEI). A multivariate analysis and multinomial regression showed a relationship between the two variables; the probability of being a victim of school bullying was 5.14 times higher among pupils with low clarity, 2.72 times higher among pupils with low repair, and 2.62 times higher among pupils with excessive attention. The results demonstrated that the better their emotional regulation and understanding, the less likely pupils are to be victims of school bullying. This confirmed that adequate emotional attention and excellent emotional clarity and repair are protective factors against victimization.

## 1. Introduction

Bullying is acknowledged around the world as a serious problem in educational settings during childhood and adolescence [1]. Due to its high prevalence, bullying is an important topic of study. According to national and international epidemiological studies, 2–12% of schoolchildren worldwide are victims of bullying [2], while in Spain, the figure is 9.3% [3].

This phenomenon is defined as a form of abuse perpetrated by peers repeatedly over time [4,5] and is characterized by a constant abuse of power, as bullies engage in deliberate acts to harm victims who find it hard to defend themselves [6,7].

School bullying gives rise to two profiles: an aggression profile and a victimization profile. The aggression profile is determined by students building an aggressive and generally violent relational dynamic toward those they see as weak, while the victims are usually the target of hostile attacks without any incitement, systematically suffering the situation with a great degree of emotional intensity that can lead to severe states of anxiety or depression and social isolation [8,9]. A perverse relationship of extended domination and submission emerges between the two profiles, leading to processes of victimization [10]. Victimization is defined as the experience of being targeted with physical, verbal, and psychological abuse by peers in the school environment on a recurrent basis [11,12].

Adequate interaction with peers in a school setting is usually a source of emotional support [13], but pupils who find themselves caught up in a process of victimization perceive this setting to be unsafe [14]. Victimization can have serious consequences on children’s development, both at the time and in the longer term [15,16], causing personal, social, and emotional problems that can lead to a deterioration in quality of life and wellbeing [5,17]. Some of the most common outcomes of victimization are sleep disorders, sadness, low self-esteem, low emotional and social self-concept, low assertiveness, negative self-perception, and increased anxiety [18,19,20,21]. Among the most serious consequences of victimization are phobias, fear, self-harm, suicidal ideation, and physical harm [20].

Previous research has identified a series of predictors of these adverse effects. One study found that the main predictors of being a victim of bullying at primary school were being a boy, being younger, exhibiting internalizing and externalizing problems, having poor academic performance, having parents with a low socioeconomic status and a low education level, and having little family support and affectivity [22,23,24,25].

However, not all victims experience negative consequences: some children respond better to bullying due to the presence of certain protective factors such as resilience (determined by high levels of emotional intelligence), family support, and support from their peers [26]. Therefore, the degree of negative psychological impact on the victim depends on the personal resources at their disposal [25,27]. One of the protective factors against school bullying is emotional intelligence [28].

Although the concept of emotional intelligence has been significantly developed, it is not free from controversy. There are two different concepts for emotional intelligence; one considers it a combination of a series of attributes related to personality [29], the other defines it as the ability to perceive and understand emotional information. Within the latter, one of the most accepted models is that of Salovey and Mayer [30]. This says that emotional intelligence is defined as people’s ability to control emotions in themselves and in others, to distinguish between different emotions, and to use this information to guide their thoughts and actions [30]. Emotional intelligence involves emotional attention (consciousness of one’s emotions and the ability to recognize these emotions and to understand their meaning), emotional clarity (the ability to understand and perceive one’s emotions), emotional repair (the ability to regulate and control positive and negative emotions), and the three processes of emotional perception, understanding, and regulation, which allow negative emotions to be minimized and controlled or inhibited and positive emotions to be prolonged, thereby intensifying both positive and negative emotions [31,32,33].

Salovey and Mayer’s ability model [27,30] considers emotional intelligence as a set of mental abilities that handle emotions and the processing of emotional information, and it studies the abilities required for this processing [32]. The way people process emotional information in stressful situations is relevant to their psychological wellbeing and adequate psychosocial development [34,35,36]. Emotions play a central role in children’s development. Emotional self-regulation in childhood can help to explain social adjustment at later stages of life: low levels of self-regulation are associated with poorer social competence [37].

Emotional intelligence in interactions with peers is considered essential for good social adaptation and positive, mature personal relationships [38,39]. Several scientific studies have linked emotional intelligence as a form of prevention to school bullying [16], where children’s ability to manage their emotions acts as a protective factor.

Some studies have focused on emotional processes as regulators and mechanisms for controlling aggressive behavior and victimization among peers [40]. Emotional regulation and emotional expression may be predictors of victimization [41,42,43]. Pupils with higher levels of emotional intelligence are less victimized by their peers and experience more positive social behavior [44,45].

Studies analyzing emotional intelligence in the context of school bullying have shown that students who suffer a great deal of intimidating behavior or bullying present low levels of emotional intelligence [16]. The findings of some research studies demonstrate that among those involved in school bullying, it is the victims who display less emotional clarity, repair, and regulation, and who need greater emotional attention [38,46,47,48,49,50].

### The Present Study

In the context of these considerations, this study focused on the phenomenon of school bullying and its relationship with self-perceived emotional intelligence. Drawing on previous research linking a lack of emotional intelligence with bullying, the main aim of the study was to analyze the mediating effect of the dimensions of emotional intelligence—attention, clarity, and repair—in relation to school victimization. As factors of protection against victimization, we expect to feel emotions and express feelings without hypervigilance (adequate emotional attention), to know and understand our emotions, to be able to distinguish one from another, to understand how they evolve and to integrate them into our thinking (excellent emotional clarity), and to regulate and control positive and negative emotions, moderating or intensifying them at our convenience (excellent emotional repair). 

## 2. Materials and Methods

### 2.1. Participants

The participants were among 20,000 pupils enrolled in years 5 and 6 at primary schools in the autonomous community of Extremadura, Spain, during the 2018–2019 school year, considering a sampling error of 3.5% and a confidence level of 96%. The total sample was made up of 822 primary school pupils from 10 public and semi-private schools, selected using intentional or convenience sampling; 45% of the sample of primary school pupils were in year 5, and 55% were in year 6. The age range was 10–12 years (M = 10.58; SD = 0.663); 46% were female and 54% male.

### 2.2. Instruments

#### 2.2.1. School Coexistence Questionnaire (SCQ), Spanish Ombudsman, 2006 

The SCQ [51] is a self-administered questionnaire that analyzes the incidence of school bullying from three perspectives: the observer, the aggressor, and the victim. Each scale includes 13 harassing behaviors classified into six modalities of bullying. The victim scale in the questionnaire requires participants to indicate whether they have experienced any of 13 different bullying scenarios since the school year began on a Likert-type scale with four response options ranging from 1 (“never”) to (“4 always”). The scenarios are: being ignored, being excluded from participating, being insulted, being called offensive or humiliating nicknames, being spoken badly of, having belongings hidden, having belongings broken, having belongings stolen, being hit, being threatened, being sexually harassed, being forced to do things they do not want to do using threats, and being threatened with weapons. Based on the direct scores from the victim scale, three categories or levels were obtained using a frequency criterion: no victimization (M ≤ 14), occasional victimization (range from M > 14 to M ≤ 17), and victimization (M > 17). 

#### 2.2.2. Trait Meta-Mood Scale-24 (TMMS-24)

To evaluate self-perceived emotional intelligence, the Spanish version of the Trait Meta-Mood Scale-24 (TMMS-24) questionnaire was used [52]. The questionnaire features 24 items, with a Likert-type scale offering five response options (1 = strongly disagree, 5 = strongly agree). It assesses three different dimensions (eight items per dimension): attention (ability to have and express feelings adequately), clarity (understanding emotional states), and repair (adequate emotional regulation).

The authors of the questionnaire classified the scores for the different dimensions in three categories: low, adequate, and excessive or excellent, with different cut-off points for boys and girls. In the emotional attention dimension, the cut-off points are: low attention (boys ≤ 21, girls ≤ 24), adequate attention (boys ≥ 22 to ≤32, girls ≥ 25 to ≤35), and excessive attention (boys ≥ 33, girls ≥ 36). In the emotional clarity dimension, the cut-off points are: low clarity (boys ≤ 25, girls ≤ 23), adequate clarity (boys ≥ 26 to ≤35, girls ≥ 24 to ≤34), and excellent clarity (boys ≥ 36, girls ≥ 35). In the emotional repair dimension they are: low repair (boys ≤ 23, girls ≤ 23), adequate repair (boys ≥ 24 to ≤35, girls ≥ 24 to ≤34), and excellent repair (boys ≥ 36, girls ≥ 35). High scores in clarity and repair indicate a good emotional intelligence in these dimensions, but this is not the case for emotional attention, as high scores can indicate an excessive vigilance of emotions and a need to improve this aspect.

### 2.3. Procedure

To carry out this research, the first step was to contact headteachers and teachers from the selected schools to explain the study objectives and to request their collaboration and permission. A consent form was then sent to parents and guardians, and the pupils who were given permission to participate were included in the study. The data collection process involved administering questionnaires by class group during school hours. The questionnaire took approximately 25 min to complete, in a quiet atmosphere to avoid pupils sharing opinions and contaminating the data. The ethical guidelines set out by the American Psychological Association were followed, ensuring the anonymity of the responses, the confidentiality of the data, and the exclusive use of this data for research purposes. The study was approved by the ethics committee of the University of Extremadura, Spain.

### 2.4. Data Analysis

Reliability analyses (Cronbach’s alpha and McDonald’s omega) and confirmatory analyses of the instruments were carried out, followed by a multivariate analysis of the mean scores in the different dimensions of emotional intelligence (attention, clarity, and repair), based on the degree of victimization (no victimization, occasional victimization, and victimization). Finally, as the authors of the TMMS-24 established three categories (low, adequate, excessive/excellent) for the different dimensions of emotional intelligence, and the victim scale includes three degrees of victimization based on frequency, it was necessary to conduct a multinomial logistic regression to establish the degree of association between the study variables, calculating the odds ratios and the respective 95% confidence intervals. Statistical analysis was carried out using the SPSS 21.0 (IBM Corp, New York, NY, USA, 2012) package for PC and JASP Free.

## 3. Results

### 3.1. Reliability and Validity Analysis of the Instruments Used in the Study

The victim scale exhibited good internal consistency, with Cronbach’s alpha (α = 0.84) and McDonald’s omega (Ω = 0.856). With respect to the TMMS-24, the reliability of each component was: attention (α = 0.79; Ω = 0.79); clarity (α = 0.71; Ω = 0.70); repair (α = 0.80; Ω = 0.81). To determine whether the models found in the original validation study of the questionnaires properly adjusted to our data, we used GFIs (goodness-of-fit indexes) (Table 1). The adjustment indexes were close to the desirable values, showing evidence of reliability and validity. Although the authors of the TMMS-24 did not validate the instrument with subjects under 12 years old, the tests confirmed reliability and validity for children up to 11.

### 3.2. Analysis of the TMMS-24 Factors Based on Degree of Victimization and Gender

A correlation analysis of the variables in the study was carried out. Table 2 shows the directly significant correlations between the victim and emotional attention variables and inverse significant correlations with clarity and emotional repair variables. All three factors of the TMMS-24 were directly significantly correlated.

Multivariate comparison of the mean scores in the attention, clarity, and emotional repair dimensions of the TMMS-24 was carried out based on the degree of victimization (no victimization, occasional victimization, and victimization), gender, and the interaction between the two variables (Table 3).

The multivariate analysis (MANOVA) showed a significant main effect of the degree of victimization (Wilks *λ* = 0.954, F(6, 1624) = 6.472, *p* < 0.001, *η* = 0.023), but no significant main effect was found for gender (Wilks *λ* = 0.999, F(3, 812) = 0.198, *p* = 0.898, *η* = 0.001) or the interaction between the degree of victimization and gender (Wilks *λ* = 0.986, F(6, 1624) = 1.904, *p* = 0.077, *η* = 0.007).

Univariate contrasts in the degree of victimization showed significant differences in emotional clarity (F(2, 814) = 5.853. *p* = 0.003, *η* = 0.014) and emotional repair (F(2, 814) = 10.895, *p* < 0.001. *η* = 0.026). No significant differences were found in emotional attention (F(2, 814) = 1.647. *p* = 0.193. *η* = 0.004). Bonferroni pairwise comparisons showed that: (1) participants in the no victimization category scored significantly higher in emotional clarity than those in the occasional victimization (*p* = 0.017) and victimization (*p* = 0.001) categories; (2) participants in the no victimization category scored significantly higher in emotional repair than those in the occasional victimization (*p* = 0.003) and victimization (*p* < 0.001) categories, while those in the occasional victimization category scored higher than those in the victimization category (*p* = 0.027).

### 3.3. Emotional Intelligence and Victimization: Multinomial Logistic Regression Analysis and Odds Ratios (ORs)

A multinomial logistic regression analysis was performed to determine which TMMS-24 variables predict victimization in school bullying. The analysis used the TMMS-24 dimensions of attention, clarity, and repair, grouped into three categories as predictor variables, and the degree of victimization, grouped into no victimization, occasional victimization, and victimization, as the dependent variable. The gender of the participants was included in the model as a control variable.

The multinomial regression analysis showed satisfactory fit (χ^2^ = 83.304(14), *p* < 0.001; χ^2^ Pearson = 135.069(70), *p* = 0.097; R Nagelkerke = 0.161), allowing 58% of the cases to be classified correctly.

Examining the results of the model with the no victimization reference category in detail, the estimates of the parameters showed that excessive attention (Wald = 6.514, *p* = 0.001), adequate attention (Wald = 11.990, *p* = 0.001), low clarity (Wald = 14.733, *p* < 0.001), adequate clarity (Wald = 11.685, *p* = 0.001), and low repair (Wald = 7.045, *p* = 0.008) were significantly and directly associated with victimization, while adequate attention (Wald = 9.284, *p* = 0.001), low attention (Wald = 17.003, *p* < 0.001), and adequate clarity (Wald = 7.746, *p* = 0.005) were significantly and directly associated with occasional victimization. Meanwhile, being a boy (Wald = 5.656, *p* = 0.017) was directly associated with victimization (Table 4).

The OR estimates for the model showed that the probability of being a victim of school bullying was 2.62 times higher among pupils with excessive attention, 2.44 times higher among pupils with adequate attention, 5.14 times higher among pupils with low clarity, 3.47 times higher among pupils with adequate clarity, and 2.72 times higher among pupils with low repair (Table 4). The probability of being an occasional victim was 1.81 times higher among pupils with adequate attention, 3.43 times higher among those with low clarity, and 1.93 times higher among those with adequate clarity. A pattern emerged, showing victims of school bullying characterized by excessive attention, low clarity, and low repair. 

Finally, the predictive model estimating the parameters for the occasional victimization reference category, which allowed a comparison of the victim and occasional victim groups, showed that low repair (Wald = 7.781, *p* = 0.005) was significantly and directly associated with victimization, meaning that probability of being a victim of school bullying was 2.55 times higher among pupils with low repair. Boys were more likely to be involved in processes of victimization (Wald = 0.757 *p* < 0.001).

## 4. Discussion

To fulfill the study objectives, the reliability and validity of the instruments were evaluated. The factorial models found in the original studies of the instruments [51,52] displayed satisfactory fit with our data. The indices were close to the optimal desired values [53], showing sufficient reliability and validity.

The aim of this study was to investigate the relationship between emotional intelligence and victimization in school bullying. The results of the multivariate analysis confirmed the existence of an association between the two variables: pupils who struggled to understand and regulate their emotional states were more likely to be involved in school bullying. Other studies have indicated significant differences between those involved and those not involved in bullying, with those involved displaying lower emotional repair. Victims in particular are characterized by low clarity and repair [47].

A detailed analysis of the results confirmed the relevance of self-perceived emotional intelligence as a predictor of victimization. Typically, the lower the excessive attention and the greater the clarity and repair among pupils, the less likely they were to be victims of school bullying [41,42,43]. Intense attention to emotions and poor abilities to understand and regulate them made children more likely to fall victim to school bullying, confirming the lack of social efficacy among victims [49,50,54]. These results support existing evidence that the clarity and repair dimensions are clearly linked [55] and that intense attention is associated with low clarity: the more focused the mind is on a problem, the less it is able to understand emotions and to seek effective solutions [56], with a negative impact on the ability to resolve conflicts with others. Victims of school bullying have a more negative social and emotional perception of themselves.

Other studies have also revealed associations between emotional intelligence and interpersonal relationships. Low emotional intelligence scores have been positively correlated with low scores in empathy, self-control in social situations, social skills, cooperation with peers, and affective relationships with others [57], while low levels of clarity and emotional repair have been found to be associated with greater social anxiety, lower empathy, and lower interpersonal satisfaction [58].

Our findings raised an interesting question: Why does clarity have stronger predictive power than emotional repair or attention? The results showed that the probability of being a victim of school bullying was 5.14 times higher among pupils with low clarity, 2.72 times higher among pupils with low repair, and 2.62 times higher among pupils with excessive attention. A limited ability to understand emotions makes it more difficult to interpret others’ intentions, potentially giving rise to errors in assessing certain behaviors [59]. This goes some way to explaining why low clarity had the greatest predictive power.

Finally, our results showed that being a boy is directly associated with victimization, i.e., boys are more likely to be involved in processes of victimization than girls. More boys than girls said they had been victims of verbal aggression, hitting, threats, and blackmail. Our findings echo those of other studies, which indicate that boys are more frequently involved in bullying as victims [60,61,62,63,64,65]. 

Some studies have shown that women are more sensitive than men. Generally, women are also more expressive than men. Some authors [66,67] have demonstrated that female gender are more perceptive, show greater empathy, and are better at recognizing others’ emotions, which explains their more positive social interactions and lesser involvement in school bullying.

### Limitations and Future Research

This study has several important limitations, including the use of self-reports as a data collection method for evaluating emotional intelligence. Pupils may have underestimated or exaggerated their perceptions of their emotional competence when completing the self-reports. Other tools for measuring emotional skills are available, which evaluate participants’ ability to complete a task [68]. When assessing school bullying, it is important to include other informants besides those directly involved. We believe that teachers are particularly well placed to analyze victimization behaviors, as they can observe and evaluate them on a daily basis in the classroom. Although the authors of the TMMS-24 did not validate the instrument with children aged under 12, the tests carried out in this study confirmed its reliability and validity for use with children aged up to 11. Other limitations include the cross-sectional design, which makes it difficult to establish further inferences about the relationship between the study variables. Finally, cultural considerations mean that caution should be exercised when extrapolating the results to other countries, especially those outside the Western world.

## 5. Conclusions

This study has made a valuable contribution to understanding the links between emotional intelligence and school bullying. The findings showed that the lower their excessive attention and the higher their emotional regulation and understanding, the less likely pupils are to be victims of school bullying. This confirms that adequate emotional attention and excellent emotional clarity and repair are protective factors against victimization.

Bullying at school is a topic of great social concern and the source of significant financial costs to the public health system. With the relevance of emotional intelligence skills as a protective factor, we recommend that emotional education is included in any actions taken to prevent or reduce school bullying. Schools are ideal settings for encouraging the development of emotional skills to allow pupils to manage their feelings and emotions, as well as those of others, and to respond appropriately to bullying [69]. As school gives children the opportunity to interact with their peers, it is the ideal setting to develop emotional abilities in a practical way by group dynamics, role-playing, communication, stimulation games, and cooperative learning. To introduce emotional education in school, a movement has arisen demanding the inclusion of social and emotional aspects as a solution to some of the most urgent problems in the education system. Emotional intelligence development programs entitled “Social and Emotional Aspects of Learning” have been implemented. They train basic abilities directly related to EI, such as emotional perception, emotional understanding, or emotional regulation, and broader and superior order aspects such as personality, self-esteem, perseverance, assertiveness, and optimism [70].

## Figures and Tables

**Table 1:** Goodness-of-fit indexes proposed for the instruments used in School Coexistence Questionnaire (SCQ) (victim scale) and the Trait Meta-Mood Scale-24 (TMMS-24).

Instruments	χ^2^	χ^2^/*df*	TLI	CFI	RMSEA
**SCQ (victim scale)**	373.415	8.486	0.843	0.847	0.096
**TMMS-24**	680.007	3.063	0.893	0.914	0.050

χ^2^ = chi-square; χ^2^/df = chi-square over degrees of freedom; TLI = Tucker–Lewis index; CFI = comparative fit index; RMSEA = root mean square error of approximation.

**Table 2:** Results for the correlation analysis of the variables under study.

Variables	1	2	3	4
1. Victim	-	0.180 *	−0.221 **	−0.285 **
2. Attention		-	0.404 **	0.352 **
3. Clarity			-	0.536 **
4. Repair				-

* Correlation is significant at the 0.05 level (bilateral). ** Correlation is significant at the 0.01 level (bilateral).

**Table 3:** Descriptive statistics for the TMMS-24 factors by degree of victimization and gender.

TMMS-24	Gender	No Victimization		Occasional Victimization		Victimization		Total	
		M	SD	M	SD	M	SD	M	SD
Attention	Girls	24.44	6.61	25.90	6.26	26.56	6.45	25.56	6.42
	Boys	26.05	7.14	24.88	5.81	26.25	6.67	25.54	6.42
	Total	25.25	6.92	25.39	6.05	26.35	6.59	25.55	6.41
Clarity	Girls	29.49	7.29	27.89	8.69	28.16	6.28	28.39	8.03
	Boys	30.52	6.43	28.89	5.67	27.05	6.84	28.81	6.30
	Total	30.01	6.87	28.39	7.35	27.38	6.68	28.62	7.14
Repair	Girls	31.13	7.11	30.05	6.65	28.84	7.51	30.20	6.92
	Boys	32.12	6.78	29.61	5.62	28.02	6.89	29.82	6.44
	Total	31.63	6.95	29.83	6.15	28.26	7.07	30.00	6.66

**Table 4:** Multinomial logistic regression with the no victimization reference category.

	Victim ^1^				Occasional Victim ^1^			
	B	OR	95% CI		B	OR	95% CI	
Excessive attention ^2^	0.962 **	2.62	1.250	5.480	0.239	1.27	0.686	2.352
Adequate attention ^2^	0.894 **	2.44	1.474	4.054	0.594 **	1.81	1.236	2.654
Low clarity ^3^	1.637 **	5.14	2.228	11.86	1.234 **	3.43	1.912	6.173
Adequate clarity ^3^	1.246 **	3.47	1.701	7.098	0.657 *	1.93	1.215	3.066
Low repair ^4^	0.999 *	2.72	1.299	5.682	0.064	1.06	0.582	1.951
Adequate repair ^4^	0.531	1.70	0.986	2.934	0.361	1.43	0.959	2.144
Girls ^5^	−0.549 *	0.577	0.367	0.908	0.208	1.23	0.866	1.750

Reference categories: No victimization ^1^. Comparison groups for emotional intelligence variable: low attention ^2^, excellent clarity ^3^, and excellent repair ^4^. Comparison groups for gender variable: boys ^5^. ** *p* < 0.001, * *p* < 0.05.
